# Orbital Plasmacytoma as the Initial Presentation of Multiple Myeloma: A Case Report

**DOI:** 10.7759/cureus.102225

**Published:** 2026-01-24

**Authors:** Anais Panossian, Timmer Verhaegh, Aram A Namavar

**Affiliations:** 1 Internal Medicine, University of California, Los Angeles, USA; 2 Internal Medicine, University of California, Los Angeles (UCLA) Health, Los Angeles, USA

**Keywords:** extramedullary plasmacytoma, multiple myeloma, orbital mass, orbital plasmacytoma, plasma cell dyscrasia

## Abstract

Multiple myeloma is a plasma cell dyscrasia that can lead to bone, renal, and hematologic complications. While multiple myeloma often manifests as a systemic disease, extramedullary manifestations can also occur in more advanced or aggressive forms of the disease. One of these manifestations is orbital plasmacytomas, which are rare and can often mimic other orbital pathologies, complicating diagnosis and treatment. This case report discusses a unique presentation of orbital plasmacytoma, emphasizing the importance of early diagnosis and tailored treatment.

## Introduction

Multiple myeloma (MM) is the second most common hematologic malignancy. It is characterized by clonal proliferation of plasma cells in the bone marrow, with the potential to cause bone lesions, renal dysfunction, anemia, and hypercalcemia [1]. MM is commonly diagnosed in adults who can be asymptomatic or present with non-specific symptoms such as fever, night sweats, weight loss, symptoms of hypercalcemia, and bone pain. A less common, aggressive type of MM is known as extramedullary MM and involves any evidence of clonal cells depositing in places outside of the bone marrow, such as soft tissues [2]. Extramedullary plasmacytomas (EMP) represent nearly 3% of plasma cell tumors, are male predominant, and commonly involve the head and neck soft tissues [3]. A rare form of EMP, orbital plasmacytoma, has primarily been reported in association with MM, as the disease in isolation is rare [4]. Orbital involvement may signal either systemic involvement of MM or recurrence of the disease. It may present with nonspecific ophthalmologic symptoms such as proptosis, diplopia, and periorbital swelling. Lesions are unilateral and commonly infiltrate the superotemporal orbit and extraocular muscles, often mimicking other orbital pathologies, including sarcomas or inflammatory conditions [5-8]. Diagnosis involves imaging the orbit with CT or MRI to characterize the extent of the lesion, as well as biopsy to confirm and distinguish the lesion from other orbital tumors [5-9]. Treatment depends on whether the disease is solitary or associated with systemic myeloma. Radiotherapy is considered first-line treatment in solitary EMP, whereas chemotherapy combined with radiotherapy is considered for systemic disease [7-9]. Here, we present an unusual case of MM manifesting as an isolated orbital plasmacytoma.

## Case presentation

A 71-year-old male with a history of bipolar disorder and major depressive disorder was transferred from an outside hospital for further evaluation of a progressive left orbital mass. Six weeks prior to admission, he noted a gradual onset of left periorbital swelling, proptosis, and diplopia. Initially attributing the swelling to eyelid edema, he self-treated with over-the-counter remedies but developed worsening double vision, prompting ophthalmologic evaluation. Laboratory studies revealed a hemoglobin level of 5.8 g/dL, and orbital imaging at the outside facility showed a destructive left orbital lesion concerning for malignancy. He received one unit of packed red blood cells prior to transfer.

On arrival, his hemoglobin was 6.6 g/dL, and he remained hemodynamically stable. Physical exam was notable for marked left-sided proptosis, chemosis, and restricted extraocular movements, especially with upward gaze, resulting in binocular diplopia. Intraocular pressure was elevated, but pupils and fundoscopic examination were otherwise unremarkable. Visual acuity was mildly reduced in the affected eye.

**Figure 1:**
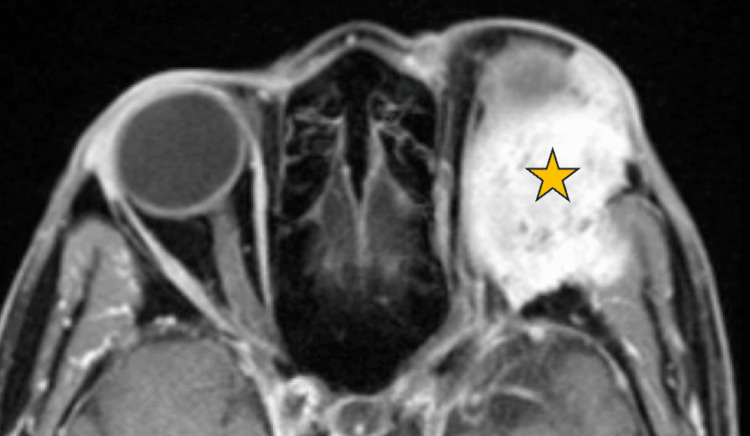
Axial T1-weighted magnetic resonance image (MRI) with contrast of bilateral orbits. The MRI demonstrates a large, destructive left spheno-orbital mass (gold star) extending into the anterior cranial fossa. The lesion produces marked proptosis and displacement of the left globe.

**Figure 2:**
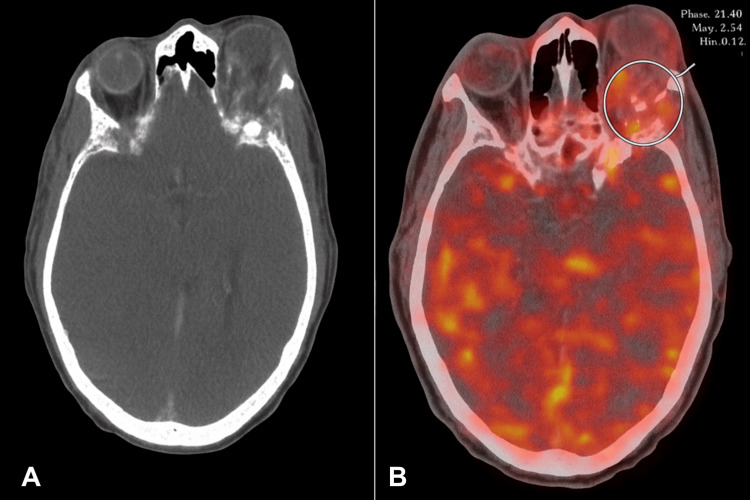
Axial CT (A) and fused PET/CT (B) correlating a destructive left spheno-orbital lesion with FDG avidity. The non-contrast axial computed tomography (CT) on the left reveals a destructive, expansile lesion involving the left spheno-orbital region. The fused positron emission tomography/computed tomography (PET/CT) on the right highlights marked fluorodeoxyglucose (FDG) uptake within the lesion (circled), confirming its hypermetabolic nature and supporting intracranial extension.

**Figure 3:**
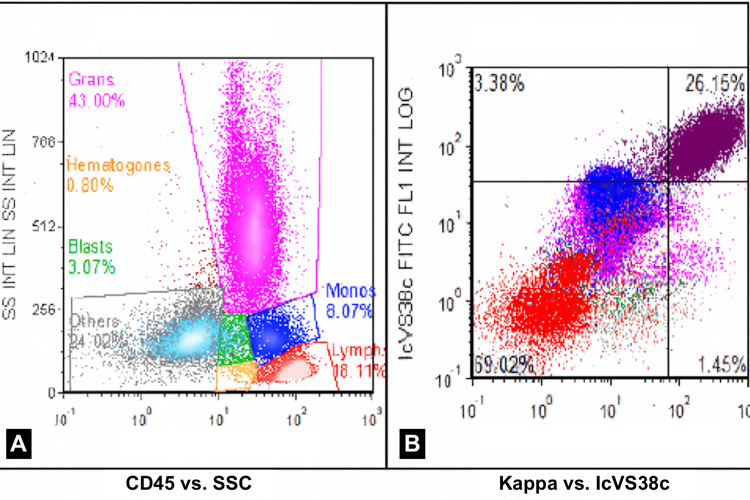
Flow cytometry demonstrating a monotypic kappa-restricted plasma cell population. Multiparametric flow cytometry of the bone marrow reveals an expanded population of plasma cells (27% of total events) with bright CD38 and CD138 expression and marked kappa light-chain restriction. The plasma cells exhibit aberrant expression of CD33, CD56, and partial CD117. Findings confirm a monoclonal plasma cell proliferation consistent with systemic plasma cell myeloma.

MRI of the orbits with and without contrast demonstrated a large, destructive spheno-orbital mass extending into the left anterior cranial fossa, with significant left orbital involvement and proptosis, as demonstrated in Figure 1. Multifocal marrow replacement and enhancement were also seen throughout the calvarium. The differential diagnosis included chondrosarcoma, plasmacytoma, Langerhans cell histiocytosis, and lymphoma. Positron emission tomography/computed tomography (PET/CT) imaging revealed a hypermetabolic left spheno-orbital mass with intracranial extension (Figure 2). An incisional biopsy of the orbital mass was performed, and analysis revealed a plasma cell neoplasm, kappa light-chain restricted, with associated eosinophilic amorphous material. Immunohistochemistry was positive for CD138, confirming plasmacytic differentiation. The adjacent lateral orbital tissue biopsy showed only benign glandular elements with mild chronic inflammation and no evidence of malignancy. These findings were consistent with an orbital plasmacytoma associated with systemic plasma cell myeloma. A subsequent bone marrow biopsy confirmed kappa light chain-predominant MM with 80-90% plasma cell involvement (Figure 3).

Multidisciplinary management involved ophthalmology, hematology/oncology, radiation oncology, and neurosurgery. The patient received high-dose intravenous methylprednisolone (100 mg daily for three days), followed by an oral prednisone taper. For ocular pressure management, dorzolamide-timolol eye drops and artificial tears were initiated, and erythromycin ointment was prescribed for corneal protection. He subsequently began external beam radiation therapy to the orbit, completing a total of 15 fractions with good tolerance. Daratumumab-based systemic therapy was initiated during hospitalization. He demonstrated significant improvement in orbital swelling and motility, with marked reductions in proptosis and diplopia by the completion of radiation therapy.

At the time of discharge after a 20-day hospitalization, the patient’s hemoglobin had stabilized, his orbital findings had improved substantially, and no new visual deficits were noted. Outpatient radiation was arranged, and he was transitioned to outpatient hematology-oncology follow-up for continued systemic therapy and surveillance. At the three-month follow-up, he remained clinically stable with continued improvement in ocular symptoms and no evidence of new disease progression.

## Discussion

MM is a malignant plasma cell disorder caused by clonal proliferation in the bone marrow, leading to complications like anemia, hypercalcemia, bone lesions, and renal dysfunction. The disease classically remains in the marrow; however, with a more aggressive clinical course, extramedullary manifestations can occur [1].

Orbital plasmacytoma is an extramedullary manifestation of MM reported in ~1% of MM cases. Orbital plasmacytomas can be diagnostically challenging due to their atypical location and initial clinical features, which can mimic other orbital neoplasms such as chondrosarcoma, lymphoma, or idiopathic inflammatory disease [2,6-8]. The patient’s symptoms - progressive orbital swelling, proptosis, and diplopia - are more commonly associated with inflammatory or structural orbital pathologies, making the diagnosis of plasmacytoma particularly elusive. This suggests that it is important to consider plasma cell dyscrasias in the differential diagnosis of orbital masses, particularly in patients with cytopenias [7,8].

This patient’s severe anemia (hemoglobin 5.8 g/dL) complicated the diagnostic process and prompted a broader systemic evaluation, including a bone marrow biopsy that showed widespread plasma cell infiltration (80-90%) of the marrow. A histopathologic study was important in this case to distinguish orbital plasmacytoma from other orbital tumors and appropriately guide treatment.

The combination of high-dose corticosteroids, localized radiation therapy, and daratumumab leads to rapid clinical improvement and underscores the importance of early intervention and the efficacy of targeted therapy in plasma cell neoplasms. This multimodal treatment approach is consistent with recommendations for extramedullary MM, where systemic treatment is more effective than local therapy, particularly for reducing the risk of relapse [1,2].

This case is unique in its presentation, diagnostic complexity, and therapeutic response. It highlights the need for heightened clinical suspicion for plasma cell disorders in patients with atypical orbital masses and systemic symptoms. Further, it underscores the value of multidisciplinary collaboration among different specialties in medicine for the management of a complex and rare presentation of MM.

## Conclusions

Orbital plasmacytoma is a rare extramedullary manifestation of MM that can mimic other orbital neoplasms. This case underscores the importance of maintaining a broad differential diagnosis in patients presenting with rapidly progressive proptosis or orbital masses. Early tissue biopsy remains essential for establishing a definitive diagnosis and distinguishing plasmacytoma from other malignant or inflammatory lesions. Prompt multidisciplinary collaboration among ophthalmology, radiology, hematology-oncology, and radiation oncology is critical to guide timely intervention. In this case, combined corticosteroid therapy, radiation, and systemic chemotherapy were associated with significant improvements in orbital symptoms and prevented irreversible vision loss. Recognition of this atypical presentation facilitates earlier diagnosis of systemic myeloma and may ultimately improve both visual and systemic outcomes.
